# 

**Published:** 1910-09

**Authors:** 


### BOOKS RECEIVED

---

The Practical Medicine Series. Ten volumes. Under the general editorial charge of Gustavus P. Head, M.D., Professor of Laryngology and Rhinology in the Chicago Post-Graduate Medical School. Vol. IV, Gynecology. Edited by Emilius C. Dudley, M.D., and C. von Bachelle, M.D. Series 1910. Chicago: The Year Book Publishers. (Price, $1.25; entire series, $10.00.)

Essentials of Laboratory Diagnosis. By Francis Ashlen Faught, M.D., Director of the Laboratory of the Department of Clinical Medicine and Assistant to the Professor of Clinical Medicine in the Medico-Chirurgical College of Philadelphia. Second edition. 12 mo, pp. 336. Illustrated. Philadelphia: F. A. Davis Co. 1910. (Cloth, $2.00.)

Refraction and Motility of the Eye. By Ellice M. Alger, M.D., Adjunct Professor of Ophthalmology at the New York Postgraduate Medical School. Octavo, pp. With 122 illustrations. Philadelphia: F. A. Davis Co. 1910. (Cloth, $2.00.)

Textbook on the Therapeutic Action of Light. By Gorydon Eugene Rogers, M.D., formerly Demonstrator of Anatomy in the University of New York. Small octavo, pp. 323. Illustrated. Published by the author.

Transactions of the Seventh Annual Conference of State and Territorial Health Officers with the United States Public Health and Marine Hospital Service, at Washington, D. C., June 2, 1910.

Hookworm Disease. By George Dock, A.M., M.D., Professor of the Theory and Practice of Medicine, Medical Department Tulane University of Louisiana, New Orleans, and Charles C. Bass, M.D., Instructor of Clinical Microscopy and Clinical Medicine, Medical Department Tulane University of Louisiana. 250 pages, royal octavo. Fifty illustrations, including one colored plate. C. V. Mosby Company, St. Louis, Publishers. (Price, $2.50.)

Dislocations and Joint Fractures. By Frederic Jay Cotton, A.M., M.D., First Assistant Surgeon, Boston City Hospital. Octavo of 654

A Textbook of Pharmacology and Therapeutics. By Arthur R. Cushny, M.A., M.D., F.R.S., Professor of Pharmacology in the University of London. Octavo, 744 pages, with 61 engravings. Lea & Febiger, Publishers, Philadelphia and New York, 1910. (Cloth, $3.75, net.)

pages, 1201 original illustrations. Philadelphia and London: W. B. Saunders Company, 1910. (Cloth, $6.00; half morocco, $7.50 net prices.)

Gynecological Diagnosis. By Walter L. Burrage, A.M., M.D. (Harv.), Clinical Instructor in Gynecology in Harvard University. Octavo, pp. 672. With 207 illustrations. New York and London: D. Appleton & Co. 1910. (Cloth, $6.00.)

Textbook of Diseases of Infancy and Childhood. By Louis Fischer, M.D., Attending Physician to the Willard Parker and Riverside Hospitals of New York. Third edition. Octavo, pp. 1003. With 333 illustrations and 29 full page plates. Philadelphia: F. A. Davis Co. 1910. (Cloth, $6.50; half morocco, $8.00, net prices.)

Nephrocoloptosis. A description of the Nephrocolic ligament and its action in the causation of Nephroptosis. With the technic of the operation of Nephrocolopexy. By H. W. Longyear, M.D., Professor of Gynecology and Abdominal Surgery in the Detroit Post-Graduate Medical School; Clinical Professor of Gynecology in the Detroit College of Medicine; Gynecologist to Harper Hospital; Ex-President of the American Association of Obstetricians and Gynecologists. Octavo, pp. 251. With 88 illustrations and a colored frontispiece. Saint Louis: C. V. Mosby Co. 1910. (Cloth, $3.00.)

